# Comparative metabolomics analysis reveals high-altitude adaptations in a toad-headed viviparous lizard, *Phrynocephalus vlangalii*

**DOI:** 10.1186/s12983-023-00513-z

**Published:** 2023-11-02

**Authors:** Xuejing Zhang, Shengkang Men, Lun Jia, Xiaolong Tang, Kenneth B. Storey, Yonggang Niu, Qiang Chen

**Affiliations:** 1 School of Life Sciences, Lanzhou University, Lanzhou, 730000 Gansu China; 2 Department of Life Sciences, Dezhou University, Dezhou, 253023 Shandong China; 3 Department of Biology, Carleton University, Ottawa, ON K1S 5B6 Canada

**Keywords:** High altitude, Metabolomics, Adaptation, *Phrynocephalus vlangalii*

## Abstract

Extreme environmental conditions at high altitude, such as hypobaric hypoxia, low temperature, and strong UV radiation, pose a great challenge to the survival of animals. Although the mechanisms of adaptation to high-altitude environments have attracted much attention for native plateau species, the underlying metabolic regulation remains unclear. Here, we used a multi-platform metabolomic analysis to compare metabolic profiles of liver between high- and low-altitude populations of toad-headed lizards, *Phrynocephalus vlangalii,* from the Qinghai–Tibet Plateau. A total of 191 differential metabolites were identified, consisting of 108 up-regulated and 83 down-regulated metabolites in high-altitude lizards as compared with values for low-altitude lizards. Pathway analysis revealed that the significantly different metabolites were associated with carbohydrate metabolism, amino acid metabolism, purine metabolism, and glycerolipid metabolism. Most intermediary metabolites of glycolysis and the tricarboxylic acid cycle were not significantly altered between the two altitudes, but most free fatty acids as well as β-hydroxybutyric acid were significantly lower in the high-altitude population. This may suggest that high-altitude lizards rely more on carbohydrates as their main energy fuel rather than lipids. Higher levels of phospholipids occurred in the liver of high-altitude populations, suggesting that membrane lipids may undergo adaptive remodeling in response to low-temperature stress at high altitude. In summary, this study demonstrates that metabolic profiles differ substantially between high- and low-altitude lizard populations, and that these differential metabolites and metabolic pathways can provide new insights to reveal mechanisms of adaptation to extreme environments at high altitude.

**Supplementary Information:**

The online version contains supplementary material available at 10.1186/s12983-023-00513-z.

## Introduction

Environmental conditions change drastically along rising altitude gradients in mountainous regions, imposing conditions on animal inhabitants including hypoxia, low temperature, and strong ultraviolet (UV) radiation [1]. Elevation is a complex environmental factor and has a profound effect on the phenotype, genotype, and geographic distribution of animals. Therefore, elevation gradients are considered to be one of the most robust "natural experiments" for verifying ecological and evolutionary adaptations of organisms [1]. The Qinghai–Tibet Plateau is known as the third pole of the Earth due to its extreme environmental characteristics, and its geographical co-ordinates are between 26° 00′ N–39° 46′ N and 73° 18′ E–104° 46′ E [2]. Hence, the area has become a natural laboratory for studying biological evolution. High-altitude acclimation of ectothermic vertebrates has received extensive attention from evolutionary biologists and physiological ecologists in recent years, mainly involving multiple levels from phenotype to genotype [3–5]. To cope with intense UV radiation, low temperatures, and hypoxia stress in the high-altitude environment, ectothermic vertebrates have developed adaptive changes in skin morphology, such as the epidermis layer, epidermal capillaries, and granular glands [6]. Adaptation to a high-altitude environment is also reflected at the physiological level with changes in cardiovascular functions, such as heart rate, cardiac output, blood pressure, blood-O_2_ affinity, and hemoglobin content [7]. Genetic evidence of high-altitude adaptation has also been identified in highland-dwelling species. For instance, gene ontology (GO) categories “response to hypoxia”, “energy metabolism”, and “response to UV damage” showed an accelerated evolutionary rate in the high-altitude lizard *Phrynocephalus erythrurus* in comparison to the lowland-dwelling *P. putjatia*, which may be conducive to high-altitude adaptation [8]. *P. erythrurus* inhabits high altitude sites and has a lower mitochondrial respiration rate and lactate dehydrogenase activity, but higher activity of β-hydroxyacyl coenzyme A dehydrogenase (HOAD) than *P. przewalskii* that lives at low altitude [9]. Hence, the potential effects of altered elevation on the metabolism of ectotherms that inhabit the Qinghai–Tibetan Plateau may be important for revealing the mechanisms of physiological adaptation in extreme environments. Several previous studies have focused on metabolic rate and metabolic enzyme activities within tissues, but these lacked data on overall changes in all or most small-molecule metabolites as well as the effect of high altitude on metabolic profiles.

Metabolomics is a systems biology approach aimed to analyze the endogenous and exogenous small-molecule metabolites within cells, tissues, or biofluids and has been widely used for investigating the interplay between organisms and their surroundings [10, 11]. For instance, ^1^H-NMR-based quantitative metabolomics analysis indicated that large rearrangements of phospholipid and nitrogen metabolism and the accumulation of free amino acids occurred in the hypoxia-exposed Siberian wood frog *Rana amurensis* [12]. Moreover, UHPLC-QE Orbitrap/MS analysis showed that freezing exposure induced a substantial change in the metabolomic profiles of the Xizang plateau frog, *Nanorana parkeri* [13]. Metabolomic analysis provides opportunities to assess regulatory mechanisms of energy metabolism and to discover new biomarkers in animals responding to environmental stress [14–16]. To date, studies using metabolomics to unravel the mechanisms of ectotherm adaptation to high-altitude environments remain scarce. Moreover, such studies typically utilized only a single assay platform, whereas the use of multi-platform metabolomics analysis, such as GC–MS and LC–MS, to explore adaptation to high-altitude environments has received little or no attention among ectothermic animals.

*P. vlangalii* (Agamidae; Phrynocephalus) is one of the toad-headed viviparous lizard species that is native to the Qinghai–Tibetan Plateau, ranging from 2000 to 4600 m above sea level [17]. The wide altitudinal range of this species makes it an ideal model for investigating adaptive evolution. Previous studies of *P. vlangalii* focused on its phylogenetic relationships, morphological characteristics, behavior, life history, and thermal physiology [18–22]. A previous study showed that the composition and structure of the gut microbiota of *P. vlangalii* varied with altitude with the proportion of *Bacteroides* at the phylum level showing a remarkable increase with rising altitude whereas Verrucomicrobia at the phylum level and *Akkermansia* at the genus level decreased with altitude, respectively [23]. Such changes in gut microbiota may play an important role in high-altitude adaptation. Moreover, acclimatization and adaptation to hypoxia was explored in high- and low-altitude *P. vlangalii* and showed that hemoglobin concentration, hematocrit, heart weight to body mass, and succinate dehydrogenase activity were all markedly higher at high altitude [24]. High-altitude *P. vlangalii* can also adjust energy metabolism to adapt to seasonal environmental changes, including their preferred body temperature, standard metabolic rate, mitochondrial respiration rate, and metabolic enzyme activities [25]. However, the metabolic regulation of adaptation to high altitude is not fully understood, and therefore, assessing metabolic profiles of liver via a multi-platform metabolomic analysis could expand our understanding of the adaptive phenotypes that support life at high altitude.

**Fig. 1:**
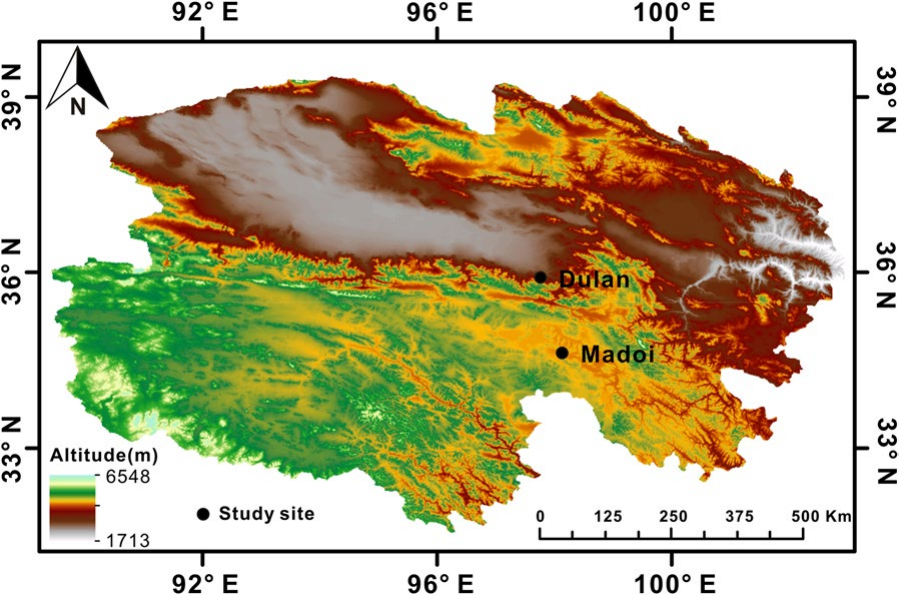
The specific locations of the two sampling sites for *P. vlangalii* lizards. The low-altitude group was collected from Dulan County (2960 m) and the high-altitude group was collected from Madoi County (4250 m) on the Qinghai–Tibet Plateau

Unfavorable conditions such as hypoxia and low temperature severely affect the survival capacity of ectotherms living at high altitude and require major adaptive changes in metabolic processes [26]. As one of the most important organs, liver serves as a hub for nutrient metabolism and excretion of metabolic waste products [27]. Thus, we hypothesized that *P. vlangalii* has developed some metabolic strategies to adapt to the extreme environment on the Qinghai–Tibetan Plateau. To test this hypothesis, we examined the hepatic metabolome of *P. vlangalii* living at high (4250 m) and low (2960 m) altitudes (Fig. 1). Our present study can provide new insights for elucidating some of the regulatory pathways and key metabolites participating in the eco-adaptations of high-altitude lizards and can contribute to an improved understanding of reptile adaptation to extreme environments.

## Results

### Overall differences in metabolite profiles of liver between high and low altitude

**Fig. 2:**
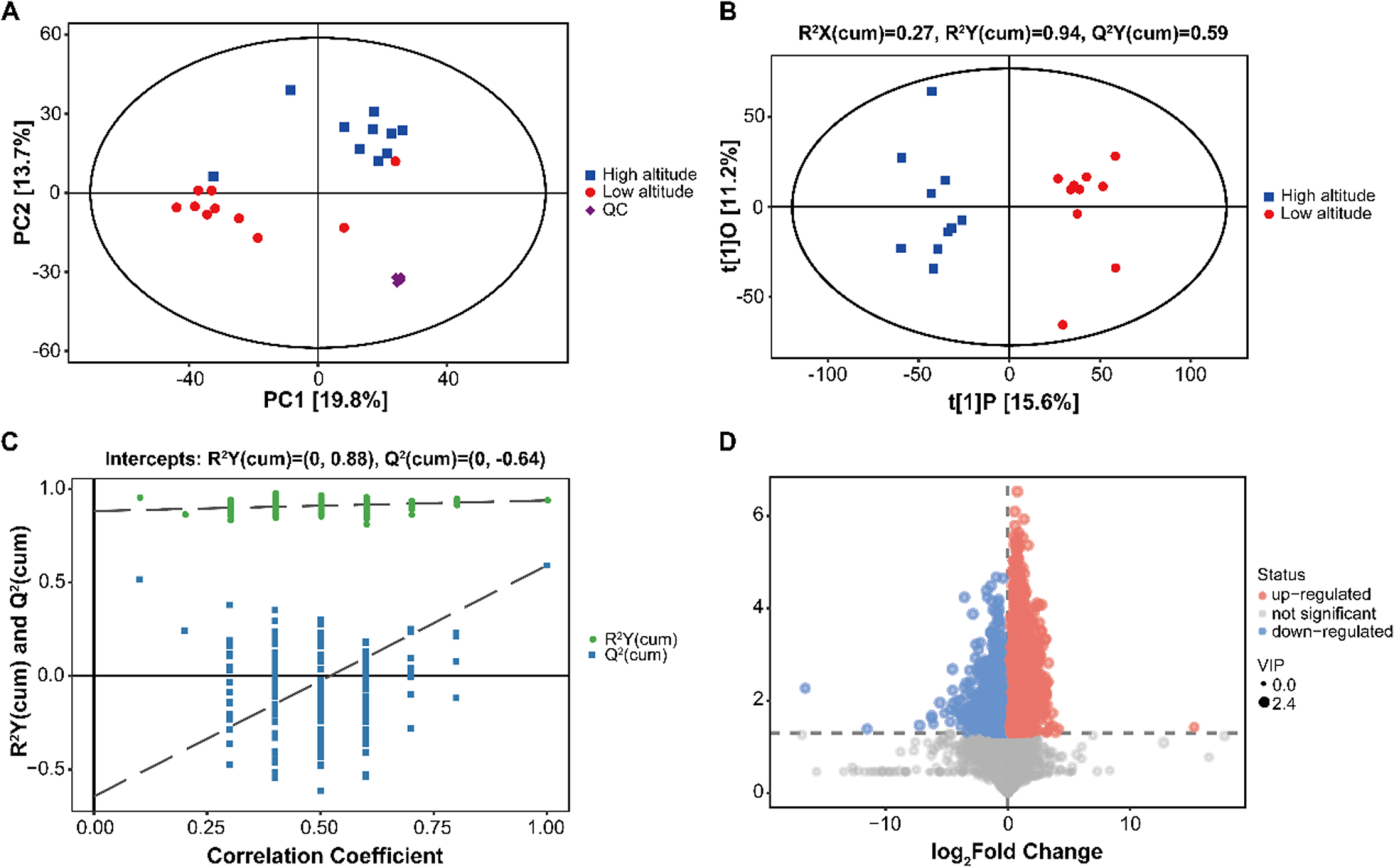
**A** Principal component analysis (PCA) score plot. Three groups are distinguished: high altitude, blue boxes; low altitude, red circles; and quality control (QC) samples, purple diamond. **B** Orthogonal projection to latent structures discriminant analysis (OPLS-DA) scores plot, showing two groups: high altitude (blue) and low altitude (red). R^2^ examines the goodness of fit whereas Q^2^ evaluates the predictive ability of the model. **C** Permutation tests for the corresponding OPLS-DA model. **D** Volcano plot showing relative changes in liver metabolites between high and low altitudes. Red circles represent metabolites that are in higher concentrations in liver of high-altitude lizards, as compared with low-altitude animals, whereas blue circles represent metabolites that are present in lower amounts in high-altitude lizards

The PCA score plot showed a dense distribution of the five QC samples and a scattered distribution between the two groups of liver samples, suggesting a good stability of the data collected by the instrument and clear differences in liver metabolites between high and low altitudes (Fig. 2A). The OPLS-DA score plot displayed a notable separation between high- and low-altitude groups, indicating that there was a significant variation in the metabolic profile of *P. vlangalii* liver between the populations from high (4250 m) and low (2960 m) altitudes and low altitudes (Fig. 2B). Q^2^ represents the predictive ability of the OPLS-DA model. R^2^ represents the ability to interpret the original data and is used to assess whether the OPLS-DA model is over-fitted. The estimated goodness of fit of R^2^Y was 94%, and the goodness of prediction of Q^2^Y was 59%, respectively. A permutation test showed that the values of R^2^Y-intercept and Q^2^-intercept were 0.88 and − 0.64, respectively (Fig. 2C). Q^2^ (cum) and R^2^ (cum) values of Y-permuted models to the left were lower than that of original models to the right and Q^2^ (cum) regression lines have a negative intercept, indicating that the OPLS-DA model is robust, reliable, and not over-fitted.

### Differential metabolites in the liver between high and low altitude

**Fig. 3:**
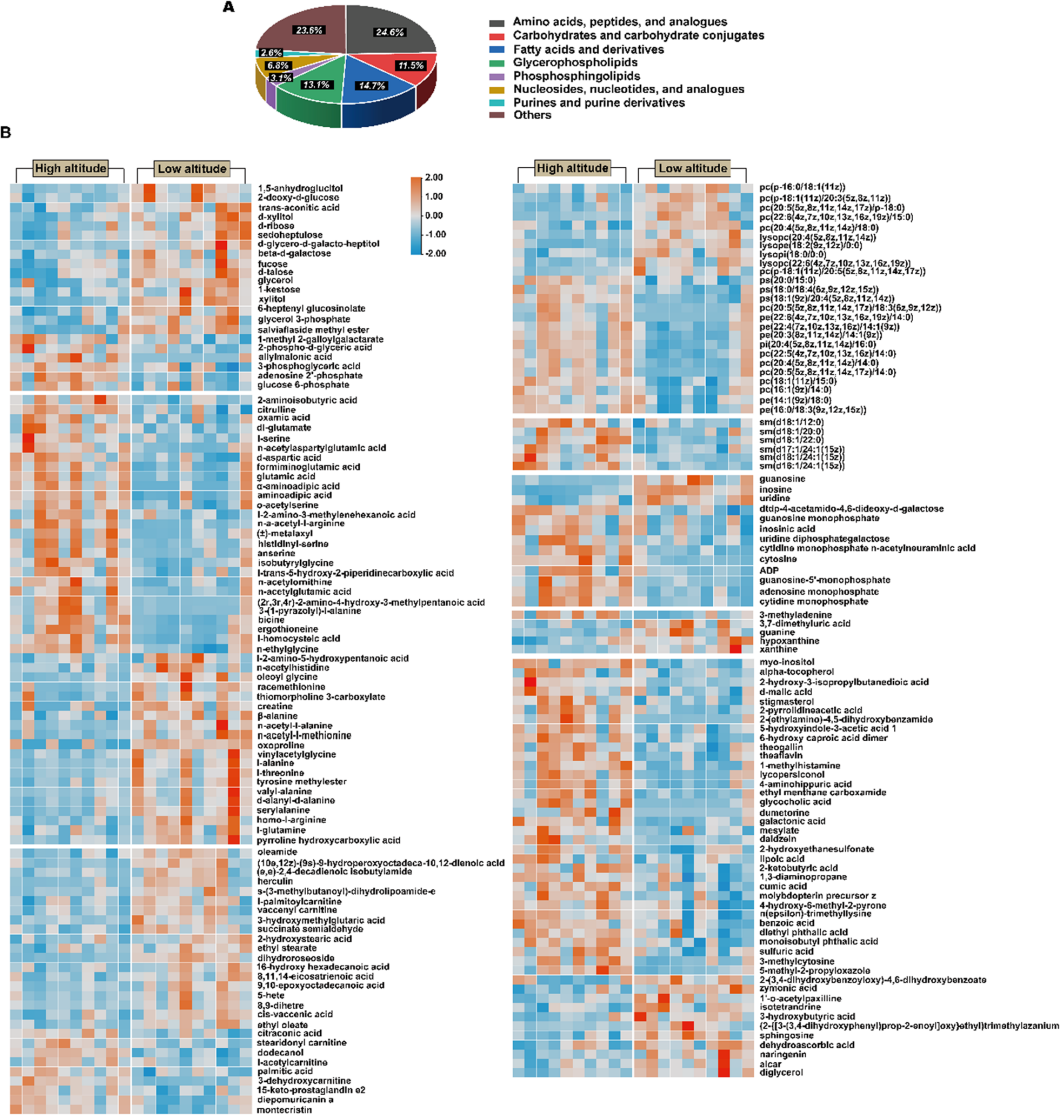
**A** Pie chart showing different classes of significantly different metabolites identified in the liver between high and low altitudes and the percentage of each class. **B** Heat map shows changes in the relative abundance of significantly different metabolites between high and low altitudes. Red color indicates high-abundance metabolites, and blue indicates low-abundance metabolites

A total of 213 differential metabolites were screened from the liver (Fig. 2D), of which 191 were identified using the mzCloud database (http://www.mzcloud.org) and an in-house MS^2^ database (Biotree Biotech Co., Ltd. Shanghai, China). These included 108 metabolites that were present in higher levels in high-altitude lizards and 83 that showed lower levels as compared to low-altitude individuals (Additional file 1: Table S1). Significantly differential metabolites in the liver between high- and low-altitude individuals were found mainly among amino acids, peptides and analogues, carbohydrates and carbohydrate conjugates, fatty acids and derivatives, glycerophospholipids, phosphosphingolipids, nucleosides, nucleotides and analogues, purines and purine derivatives as well as some others (Fig. 3A). A heat map shows the relationships and changes in relative abundance of metabolites that were significantly different between the two groups (Fig. 3B).

### Metabolic pathway analysis

**Fig. 4:**
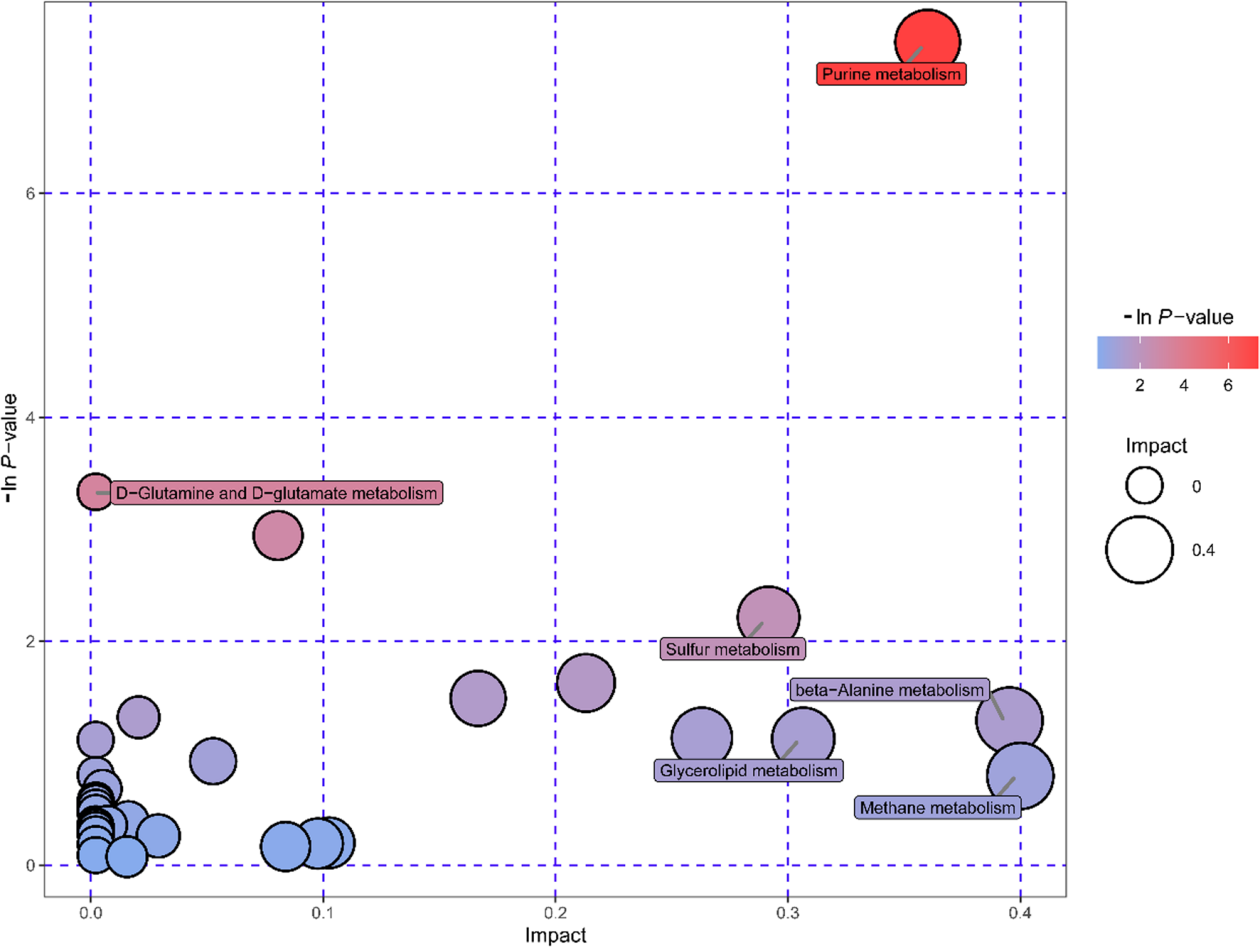
Bubble chart displays pathway analysis of significantly different metabolites in the liver between high and low altitude. Each bubble represents a metabolic pathway. The color and size of the bubbles indicate the influencing factors of the pathway in the topological analysis. A darker color (smaller *p* value, expressed as − ln(*p* value)) indicates a significantly enriched pathway, and a larger bubble (higher impact scores) indicates a more important pathway, respectively

**Fig. 5:**
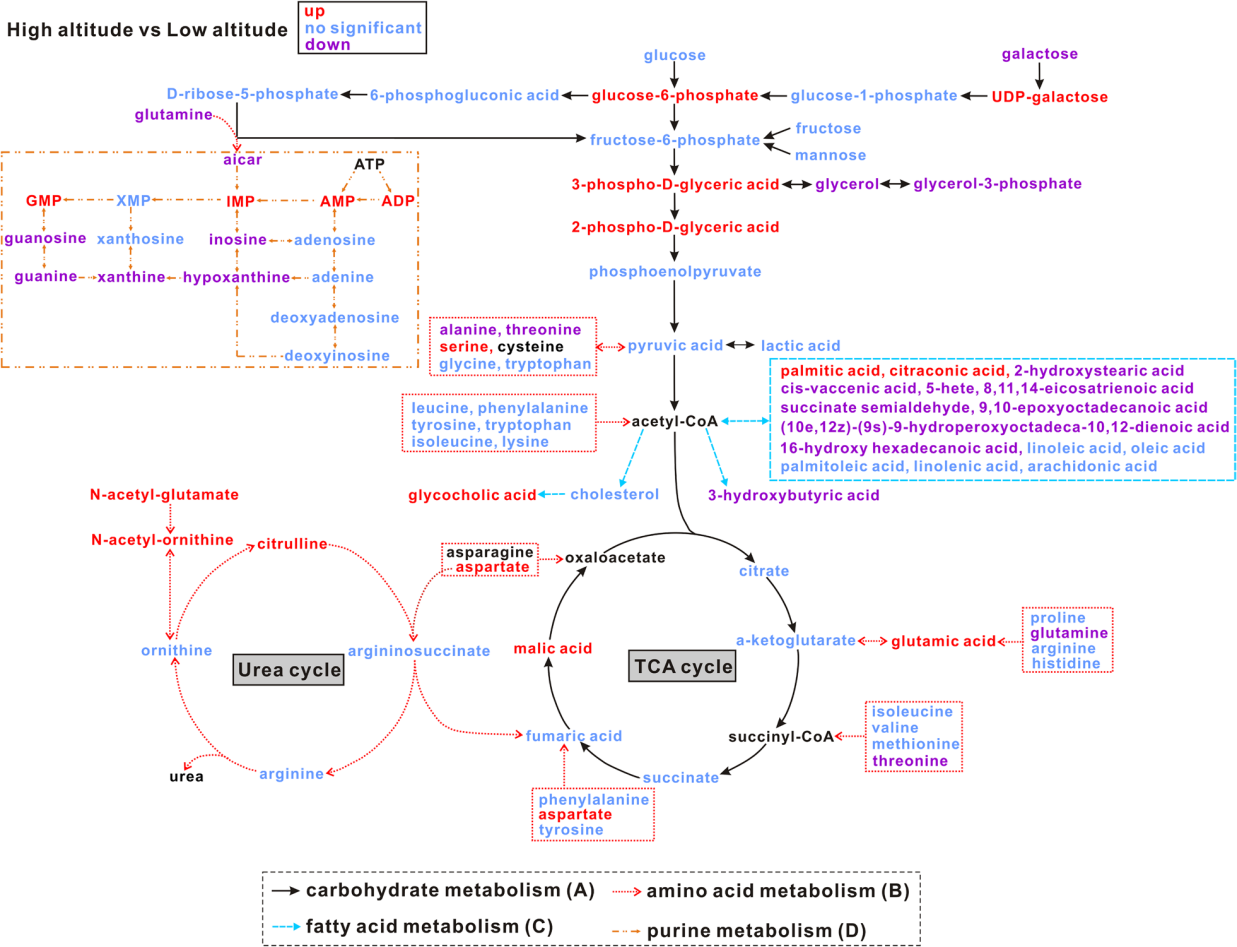
Metabolic network of the significantly different metabolites involved in **A** carbohydrate metabolism, **B** amino acid metabolism, **C** fatty acid metabolism, and **D** purine metabolism. Red indicates up-regulated metabolites, blue indicates metabolites with no significant change, and purple indicates down-regulated metabolites. Black shows metabolites that failed to be detected by metabolomics technology

Metabolic pathways that showed significant differences between high- and low-altitude lizards were visualized using a bubble chart (Fig. 4). The metabolic pathways identified were mainly those involved in amino acid metabolism including arginine and proline metabolism (ko00330), alanine, aspartate and glutamate metabolism (ko00250), histidine metabolism (ko00340), beta-alanine metabolism (ko00410), and glycine, serine and threonine metabolism (ko00260). Moreover, purine metabolism (ko00230), sulfur metabolism (ko00920), and glycerolipid metabolism (ko00561) also showed significant differences in the liver from high- versus low-altitude lizards. Differences in primary metabolites linked to major metabolic processes are shown in Fig. 5, such as carbohydrate metabolism, amino acid metabolism, fatty acid metabolism, and purine metabolism. The contents of glucose 6-phosphate, 3-phospho-D-glyceric acid, 2-phospho-D-glyceric acid, and malic acid were all significantly greater, but the contents of glycerol, glycerol 3-phosphate, and galactose in the liver were significantly lower in high-altitude lizards as compared with low-altitude individuals. Most amino acids showed no significant difference in liver between high- and low-altitude lizards. However, the levels of glutamic acid, aspartate, serine, and citrulline were higher and glutamine, alanine, and threonine were lower in high-altitude individuals. Multiple free fatty acids showed a lower level in high-altitude lizards except for palmitic acid and citraconic acid. Common unsaturated fatty acids, such as palmitoleic acid, oleic acid, linoleic acid, linolenic acid, and arachidonic acid, did not differ significantly between high- and low-altitude lizards. Moreover, the levels of hypoxanthine, inosine, xanthine, guanine, and guanosine were all lower but IMP, AMP, ADP, and GMP were higher in high-altitude lizards than those in low-altitude individuals (Fig. 5).

## Discussion

Herein, we identified and analyzed differences in liver metabolites between *P. vlangalii* lizards living at high (4250 m) versus low (2960 m) altitudes on the Tibetan Plateau using multi-platform metabolomics. Our results highlight several major differences involving carbohydrate metabolism, amino acid metabolism, purine metabolism, and glycerolipid metabolism between the two groups that may be associated with high-altitude adaptation. Similarly, a study of differential metabolites between high-altitude and low-altitude bumblebees, *Bombus pyrosoma*, showed that they were enriched in β-alanine metabolism, purine metabolism, alanine, aspartate and glutamate metabolism, glycerophospholipid metabolism, cysteine and methionine metabolism, and fatty acid degradation at high altitude [28].

### Carbohydrate metabolism

Under hypoxia, animals have to rely more on glycolysis to produce ATP with glucose processed to pyruvate instead of entering the tricarboxylic acid (TCA) cycle and with pyruvate transformed into a variety of end products (e.g. lactate, alanine, ethanol, etc.) in reactions that restore NAD^+^ [29]. Thus, maintenance of glucose supply in liver (typically from stored glycogen) is crucial for hypoxia survival of the whole animal. For instance, extreme hypoxia exposure resulted in a 1.8-fold increase in the glucose concentration in liver of the Siberian wood frog *R. amurensis* [12]. However, we found no significant differences in glucose levels between high- and low-altitude lizards. This also seems reasonable because animals living constantly at high altitude have implemented various metabolic adaptations to deal with long-term low oxygen levels, which may be different from the responses exhibited by animals transported rapidly from low to high altitudes or simply exposed to low oxygen levels. Two glycolytic intermediates (3-phospho-D-glyceric acid and 2-phospho-D-glyceric acid) showed significantly higher levels in high-altitude lizards than in low-altitude individuals, and these may be mainly derived from glycerol. Indeed, glycerol levels were significantly lower in high-altitude lizards. Lower glycerol levels may result from weak glycerophosphocholine hydrolysis, with high levels of glycerophosphocholine observed in the liver of high-altitude individuals. Significant accumulation of glucose-6-phosphate was observed in lizard liver at high altitude, but 6-phosphogluconic acid and D-ribose-5-phosphate did not differ significantly between the two populations. Therefore, the pentose phosphate pathway does not appear to be remodeled in the liver of high-altitude lizards.

Pyruvate, citrate, and ketoglutarate contents were not significantly different between high- and low-altitude lizards, indicating that pyruvate production from glycolysis continued and that the first segment of the TCA cycle (oxaloacetate + acetyl CoA → citrate ↔ isocitrate ↔ α-ketoglutarate) was not affected by the high-altitude environment. Pyruvate could be converted to lactate to regenerate NADH for continued glycolysis, but lactate did not accumulate in high-altitude lizards. This result indicated that high-altitude *P. vlangalii* relies mainly on aerobic metabolism (channeling pyruvate into the TCA cycle) rather than anaerobic metabolism. Moreover, the NAD^+^ required for the second segment (α-ketoglutarate → succinyl-CoA ↔ succinic acid) was most likely derived from a countercurrent of the third segment of TCA (oxaloacetate ↔ malate ↔ fumarate ↔ succinic acid). This interpretation of our data is well supported by the higher levels of malate in high-altitude lizards.

### Amino acids metabolism

It is generally accepted that alanine as an end product of anaerobic glycolysis and aspartate depleted by the malate-aspartate shuttle is believed to be crucial for hypoxic survival [30]. High concentrations of alanine may also result from amino acid degradation with amino groups transferred from other amino acids onto pyruvate, leaving the carbohydrate skeleton to be catabolized or recycled. However, a lower level of alanine was observed in liver of high-altitude lizards, which may be involved in glutamate regulation under hypoxic conditions, since alanine aminotransferase can transfer the amino group from alanine onto alpha-ketoglutarate to make glutamate [31]. Indeed, we found higher levels of glutamate in high-altitude individuals. In addition to glycogen, some amino acids can be used as anaerobic fuels under hypoxia/anoxia. The most notable are aspartate, asparagine, glutamate, and glutamine [32]. Higher levels of aspartate and glutamate were shown in high-altitude lizards. Aspartate and glutamate metabolism are essential for maintaining energy balance, and the unchanged level of lysine suggests that the accumulation of both amino acids in high-altitude lizards mainly stems from protein and/or amino acid catabolism, since lysine is a vital precursor for de novo synthesis of glutamate [33]. In our present study, most amino acids showed no significant differences in liver between the two groups, suggesting that the source and destination of these amino acids are in dynamic balance that is not disrupted by environmental stress at high altitude. Our results also suggested an important role for glutamate and aspartate in high-altitude adaptation in lizards.

### Purine metabolism

In hypoxia-tolerant species, elevated adenosine levels can disengage energy-intensive cellular processes [34], regulate the rate of glycolysis, and initiate metabolic suppression, thereby slowing down energy consumption while improving anaerobic ATP production [35, 36]. However, we found no significant differences in the levels of both adenine and adenosine between high- and low-altitude lizards. Moreover, ADP and AMP levels were higher in high-altitude lizards, and may be mainly due to the degradation of ATP and/or a reduced breakdown from AMP to adenosine. To some extent, cellular metabolism is regulated by a trade-off between ATP-consuming and ATP-producing pathways [37]. For example, ATP has an allosteric inhibitory effect on rate-limiting enzymes of the glycolytic pathway (e.g., phosphofructokinase), that can be overcome by AMP activation of phosphofructokinase. A slight decrease in levels of ATP can result in a large proportional increase in AMP and further result in a substantial activation of phosphofructokinase under energy stress conditions. Moreover, the rate of oxidative phosphorylation in mitochondria can be modulated via the availability of ADP. Therefore, it is not surprising that adenylate levels do not change much in most stress-tolerant animals [37].

During hypoxia, the xanthine oxidase (XO) system appears to be a main endogenous source of reactive oxygen species (ROS) [38, 39]. Hypoxanthine is derived from the breakdown of ATP and catalyzed by XO to form xanthine [40, 41]. The accumulated xanthine and hypoxanthine provide substrates for a burst of ROS production and may lead to oxidative stress. Thus, xanthine levels are considered to be an indicator of the degradation of ATP to adenosine with associated ROS production [42]. However, intermediates of the purine nucleotide catabolic pathway (hypoxanthine, xanthine, and guanine) showed lower levels in liver of high-altitude lizards than in low-altitude individuals, which was also supported by the high levels of AMP and GMP. It is known that hypoxia strongly affects mitochondrial respiration and cellular redox homeostasis, such as increasing the production of ROS owing to the electron slip from electron transport system complexes and/or reverse electron flow through mitochondrial Complex I [43, 44]. We can also speculate that high-altitude *P. vlangalii* lizards, living permanently in a situation of reduced oxygen and colder environments than their conspecifics at low altitude, may not experience high levels of oxidative stress. Such speculation is supported by the lower levels of dehydroascorbic acid in high-altitude species. This can also be supported by a previous study where malondialdehyde levels did not differ significantly and high-altitude *P. vlangalii* had lower total antioxidant capacity than low-altitude individuals, although oxidative stress levels were not directly detected [45]. Similar results were found in another lizard, *Psammodromus algirus*, and the frog *N. parkeri*, where oxidative stress levels were lower in high-altitude animals than in low-altitude individuals [46, 47]. All animals have evolved protective and repair mechanisms such as antioxidant defense systems to effectively and timely remove/degrade ROS [48]. Lipoic acid is not only an important cofactor for aerobic metabolism, but also a vitamin-like antioxidant that effectively scavenges ROS to help maintain a healthy cellular redox state [49]. Alpha-tocopherol (vitamin E) is the most common neutral lipid and functions as a non-enzymatic antioxidant to provide a first line of defense against damaging oxidative reactions on cellular membranes [50, 51]. In the present study, high-altitude lizards showed higher levels of lipoic acid and alpha-tocopherol in liver than in low-altitude individuals, which would be beneficial for rapid scavenging of ROS.

### Lipid and fatty acid metabolism

It is well known that membrane lipid reorganization refers to changes in the composition of membrane lipids, that can occur with temperature acclimation, seasonal adaptation, or evolutionary adaptation [52]. Glycerophospholipids (i.e. phospholipids with glycerol as a backbone), the most abundant lipids in animal membranes, can be classified as glycerophosphatidic acid (PA), glycerophosphocholine (PC), glycerphosphoethanolamine (PE), glycerophosphoserine (PS), glycerophosphoglycerol (PG), and glycerophosphoinositol (PI). High-altitude inhabitants live at lower mean annual temperatures than do low-altitude inhabitants, so we hypothesized that high-altitude lizards may also adopt membrane lipid restructuring as a strategy to adapt to colder temperatures at high altitude. In the present study, we found that there were significant differences in the level of 12 PCs, six of which increased and the other six decreased when comparing high- and low-altitude lizards. Five PEs showed higher levels in high-altitude lizards. These results suggested that significant differences in lipid composition exist between high- and low-altitude lizard populations, which may be crucial for adaptation to high-altitude life. Previous studies have shown that the changes in membrane lipids play an important role in modifying enzyme thermal sensitivity and activity [53, 54]. Changes in the concentrations of PCs and PEs were related to changes in phospholipid metabolism [55]. Moreover, 6 sphingolipids (the major component of cell membranes) were all significantly higher in high-altitude lizards than in low-altitude individuals. Phosphatidylinositol typically represents a low percentage of total phospholipids (usually less than 10%) and also undergoes significant restructuring, with a higher level of pi(20:4[5z,8z,11z,14z]/16:0) in high-altitude *P. vlangalii*. A higher free phospholipid content is probably needed to preserve membrane fluidity [56] and contributes to long-term cold survival of lizards at high altitudes.

The higher abundance of glucose-6-phosphate, 3-phospho-D-glyceric acid, and 2-phospho-D-glyceric acid in high-altitude lizards suggests an enhanced carbohydrate metabolism, which could lead to a suppression of lipid catabolism and a decrease in the content of fatty acids. Indeed, the levels of most fatty acids were significantly lower in high-altitude lizards than those in low-altitude individuals. Moreover, β-hydroxybutyric acid, as a ketone body, is thought to be an indicator of fatty acid metabolism, and the lower level of β-hydroxybutyric acid coincided with lower levels of fatty acids in high-altitude lizards. It has also been demonstrated in many studies that animals respond to high altitude hypoxia by reducing the fatty acid metabolism since fatty acids are catabolized only by oxygen-dependent pathways [57, 58]. The levels of two important essential fatty acids, linoleic acid and linolenic acid, did not differ significantly, suggesting that there were no differences in diet and/or the utilization of two fatty acids between the two populations. Therefore, combined with the above discussion on carbohydrate metabolism, we believe that high-altitude lizards mainly rely on aerobic metabolism with a preferential use of carbohydrate fuel, rather than lipids, as their main energy source. In support of this, glycolysis is thought to have less thermal dependence than β-lipid oxidation [59] and, therefore, it may be favorable for carbohydrates to be mobilized in low-temperature environments at high altitude. In addition, carbohydrates can be metabolized both aerobically and anaerobically, whereas fatty acid oxidation is an aerobic process only and consumes more oxygen than glycolysis [60].

## Conclusions

In summary, this study provides a comprehensive metabolic profiling of liver, a key organ for adaptation to high-altitude environments, and we identified several metabolic pathways that showed adaptive changes in *P. vlangalii*. These data contribute to explaining the molecular mechanisms of high-altitude adaptation. We demonstrated that aerobic metabolism was sustained in high-altitude lizards with carbohydrates as a probable main fuel rather than lipids. Aspartate and glutamate metabolism and phospholipid metabolism changed in high-altitude lizards and a marked remodeling of membrane lipids occurred. These metabolic regulation strategies in response to extreme stress at high altitude may be beneficial to improving the utilization of energy substrates and maintaining body homeostasis and have great significance for life at high altitude.

## Materials and methods

### Sample collection

Adult males *P. vlangalii* lizards (n = 10 at each altitude) were collected at altitudes of 2960 m (Dulan County; 36.04°N, 97.76°E) and 4250 m (Madoi County; 34.75°N, 98.13°E) above sea level during July in Qinghai province, China (Fig. 1). Body mass and snout-vent length were recorded immediately after collection (Table 1) and then lizards were quickly euthanized by decapitation near the sampling site. Liver tissue from both groups was immediately removed, frozen in liquid nitrogen, and then shipped on dry ice to Biotree Biotech Co., Ltd. (Shanghai, China) for LC–MS and GC–MS analysis.

**Table 1:** Morphometric parameters of *P. vlangalii* collected from high- and low-altitude

	Low altitude	High altitude
Mean body mass (g)	7.83 ± 0.27	8.27 ± 0.23
Mean snout-vent length (cm)	5.79 ± 0.08	5.74 ± 0.04

The data are expressed as the means ± SEM (n = 10)

### Metabolite extraction

Extraction of metabolites from liver samples followed the standard process of Biotree Biotechnology Co., Ltd. as we reported previously with a minor modification [61]. Liver samples (50 mg, n = 10 for each group) were each mixed with 1000 μL of extraction reagent (methanol:acetonitrile:water at 2:2:1; v:v:v) containing adonitol (1000:5; v:v) and then quickly homogenized using an automatic homogenizer (JXFSTPRP-24, Shanghai Jingxin Technology Co., Ltd., Shanghai, China) followed by ultrasound-treatment for 5 min (repeated 3 times) with an ultrasonic apparatus (PS-60AL, Leidebang Electronics Co., Ltd., Shenzhen, China) while samples were held in ice water. Samples were then left to stand at − 40 °C for 1 h and finally centrifuged at 13,800 g for 15 min at 4 °C. For subsequent ultra-high performance liquid chromatography coupled with Q-Exactive MS (UHPLC-QE-MS) analysis, supernatants were removed and quality control (QC) samples (n = 5) were prepared by mixing equal aliquots (10 μL) of supernatants from all samples. For gas chromatography coupled with a time-of-flight mass spectrometer (GC–TOF-MS) analysis, aliquots of 200 μL of the supernatant from each sample were transferred to new EP tubes, as well as preparing QC samples (n = 5) by mixing an equal aliquot (40 μL) of supernatants from all of the samples.

### UHPLC-QE-MS metabolomics analysis

UHPLC-QE-MS analysis was performed using an Agilent 1290 UHPLC system with a UPLC BEH Amide column (2.1 mm × 100 mm, 1.7 μm) coupled to a Q Exactive HFX mass spectrometer (Orbitrap MS, Thermo). The mobile phase A was composed of 25 mM ammonium acetate and 25 mM ammonia hydroxide (pH 9.75), and the mobile phase B was acetonitrile. The QE HFX mass spectrometer was used due to its ability to acquire full MS/MS spectra on information-dependent acquisition (IDA) mode in the control of the acquisition software (Xcalibur, Thermo). The auto-sampler temperature was 4 °C, and the injection volume was 2 μL. The ESI source conditions were set as follows: sheath gas flow rate, 30 Arb; aux gas flow rate, 25 Arb; capillary temperature, 350 °C; full MS resolution, 60,000; MS/MS resolution, 7500; collision energy, 10/30/60 in NCE mode; spray Voltage, 3.6 kV (positive ionization mode) and − 3.2 kV (negative ionization mode), respectively [62]. Raw data were converted to the mzXML format using ProteoWizard and processed with an in-house program, which was developed using R and based on XCMS (version 3.2), for peak detection, extraction, alignment, and integration. Then, metabolite annotation was conducted using an in-house MS^2^ database (BiotreeDB).

### GC–TOF-MS metabolomics analysis

Metabolite derivatization was conducted according to the following procedures. Firstly, all samples were mixed with 40 μL of methoxyamination hydrochloride (dissolved in 20 mg mL^−1^ pyridine) and incubated at 80 °C for 30 min after freeze-drying, then N,O-Bis (trimethylsilyl) trifluoroacetamide (BSTFA) with 1% trimethylchorosilane (TMCS) was added and incubated at 70 °C for 1.5 h. Finally, 5 μL of fatty acid methyl esters (FAMEs, dissolved in chloroform) was added to the QC sample, and then all samples were analyzed by GC–TOF-MS. A 7890 Agilent gas chromatograph system was coupled with a Pegasus HT time-of-flight mass spectrometer (Leco, St. Joseph, MI, USA) and a DB-5MS capillary column (30 m × 250 μm × 0.25 μm; Agilent J&W Scientific, Folsom, CA, USA). Helium was used as the carrier gas at a constant flow rate of 1 mL min^−1^. The initial temperature was maintained at 50 °C for 1 min, raised to 310 °C at a rate of 10 °C min^−1^, and then kept at 310 °C for 8 min. The injection, transfer line, and ion source temperatures were set to 280, 280, and 250 °C, respectively. The injection volume was 1 μL. The energy was − 70 eV in electron impact mode. Mass spectrometry data were acquired in full-scan mode with the m/z range of 50–500 at a rate of 12.5 spectra per second after a solvent delay of 6.35 min [63]. Raw data were pre-processed for peak extraction, baseline adjustment, deconvolution, alignment, and integration using Chroma TOF (V 4.3x, LECO) software. Metabolite identification was performed using LECO-Fiehn Rtx5 database. Moreover, the peaks detected in QC samples were filtered out according to the coefficient of variation (relative standard deviation, RSD > 30%) and detection rate (DR < 50%) [64].

### Statistical analysis

Metabolite data from GC–TOF-MS and UHPLC-QE-MS analyses were used to perform multivariate statistical analysis, including principal component analysis (PCA) and orthogonal partial least squares-discriminant analysis (OPLS-DA), with univariate statistical analysis (Student’s *t*-test) by SIMCA (V15.0.2, Sartorius Stedim Data Analytics AB, Umea, Sweden). The R^2^X, R^2^Y, and Q^2^ values were calculated to assess the quality of the analytic models, and permutation tests with 200 iterations were conducted to confirm the OPLS-DA model. Significantly different metabolites were screened according to thresholds with *P* < 0.05 from a two-tailed *t*-test and the variable importance for the projection (VIP > 1) values. Pathway analysis was performed on MetaboAnalyst 5.0 (http://www.metaboanalyst.ca) and KEGG database (http://www.kegg.jp), and significant metabolic pathways were screened based on pathway impact scores of > 0.05 and − ln(*P* values) of > 1.0.

### Supplementary Information

**Additional file 1.** **Table S1.** All significantly changed metabolites between low- and high-altitude from metabolomic analysis.

## Data Availability

Metabolomics data are available in the MetaboLights repository (http://www.ebi.ac.uk/metabolights) with accession number MTBLS6900.
